# Muscle innervation zone estimation from monopolar high-density M-waves using principal component analysis and radon transform

**DOI:** 10.3389/fphys.2023.1137146

**Published:** 2023-03-15

**Authors:** Chengjun Huang, Zhiyuan Lu, Maoqi Chen, Cliff S. Klein, Yingchun Zhang, Sheng Li, Ping Zhou

**Affiliations:** ^1^ Department of Neuroscience, Baylor College of Medicine, Houston, TX, United States; ^2^ School of Rehabilitation Science and Engineering, University of Health and Rehabilitation Sciences, Qingdao, Shandong, China; ^3^ Guangdong Work Injury Rehabilitation Center, Guangzhou, Guangdong, China; ^4^ Department of Biomedical Engineering, University of Houston, Houston, TX, United States; ^5^ Department of Physical Medicine and Rehabilitation, University of Texas Health Science Center at Houston, Houston, TX, United States; ^6^ TIRR Memorial Hermann Hospital, Houston, TX, United States

**Keywords:** innervation zone, monopolar, M wave, electrode array, principal component analysis, radon transform

## Abstract

This study examined methods for estimating the innervation zone (IZ) of a muscle using recorded monopolar high density M waves. Two IZ estimation methods based on principal component analysis (PCA) and Radon transform (RT) were examined. Experimental M waves, acquired from the biceps brachii muscles of nine healthy subjects were used as testing data sets. The performance of the two methods was evaluated by comparing their IZ estimations with manual IZ detection by experienced human operators. Compared with manual detection, the agreement rate of the estimated IZs was 83% and 63% for PCA and RT based methods, respectively, both using monopolar high density M waves. In contrast, the agreement rate was 56% for cross correlation analysis using bipolar high density M waves. The mean difference in estimated IZ location between manual detection and the tested method was 0.12 ± 0.28 inter-electrode-distance (IED) for PCA, 0.33 ± 0.41 IED for RT and 0.39 ± 0.74 IED for cross correlation-based methods. The results indicate that the PCA based method was able to automatically detect muscle IZs from monopolar M waves. Thus, PCA provides an alternative approach to estimate IZ location of voluntary or electrically-evoked muscle contractions, and may have particular value for IZ detection in patients with impaired voluntary muscle activation.

## 1 Introduction

The innervation zone (IZ) of a muscle is the region where muscle fibers are innervated by motor axon terminals. The architecture of the IZ can influence electromyographic (EMG) signal characteristics recorded from the muscle surface (Nishihara et al., 2010; Rantalainen et al., 2012; Gallina et al., 2013; Ye et al., 2015; Smith et al., 2017; de Souza et al., 2022). The ability to detect the location of the IZ using EMG techniques has implications for understanding muscle function in health and disease. Thus, monitoring changes in IZ location may provide valuable information about the processes of motor unit remodeling associated with aging, disease, and injury (Jahanmiri-Nezhad et al., 2015; Rasool et al., 2017; Dias et al., 2018; Li et al., 2021). In addition, the ability to detect the IZ has important clinical value. For example, one clinical application of IZ estimation is to guide botulinum toxin (BTX) injection more precisely for treating spasticity in patients with neurological injuries such as stroke and cerebral palsy (Van Campenhout and Molenaers, 2011; Guzmán-Venegas et al., 2014; Zhang et al., 2019; Chen et al., 2020; Zhang et al., 2021). The effectiveness of BTX treatment has been reported to depend on the distance between the injection site and the IZ (Shaari and Ira Sanders, 1993; Lapatki et al., 2011; Kaymak et al., 2018).

The IZ can be identified through EMG signals recorded by a linear electrode array or a matrix of electrodes placed over the muscle (Drost et al., 2006; Barbero et al., 2012; Piccoli et al., 2014; Campanini et al., 2022). Most investigators have estimated the location of the IZ based on surface EMG recordings of voluntary muscle contractions and processing the signals in a single differential or bipolar configuration (Ostlund et al., 2007; Mesin et al., 2009; Enck et al., 2010; Barbero et al., 2011; Beck et al., 2012; Ullah et al., 2014; Marateb et al., 2016; Liu et al., 2019; Mancebo et al., 2019; Liu et al., 2020; Zhang et al., 2020), whereas few have processed monopolar signals for IZ estimation (Rodriguez-Falces, 2017). When EMG signals are processed in a differential configuration the IZ location may correspond to either a reversal in EMG signal polarity between two adjacent channels along the muscle fibers, or the smallest amplitude in a single channel.

Although voluntary contractions are convenient for estimating the IZ, they may not be feasible in patients with significant paralysis or poor motor control. An alternative method for IZ location is to record compound muscle action potentials (or M waves) evoked by electrical stimulation of the motor nerve, but few have used this approach (Zhang et al., 2017). In two reports, the IZ location was found to be similar when based on M waves and voluntary EMG (Guzmán-Venegas et al., 2016) (Huang et al., 2019). When recording M waves using electrode arrays, a monopolar electrode configuration is often used because bipolar configuration may considerably attenuate M wave content (Tucker and Türker, 2005; Hadoush et al., 2009; Rodriguez-Falces and Place, 2018). There is a need to further develop appropriate methods to automatically estimate the IZ from M wave signals recorded in a monopolar configuration.

In this study we investigated two methods to estimate the IZ from monopolar M-wave recordings. One method was based on principal component analysis (PCA); specifically, the second principal component coefficients derived from PCA, which are related to time delays of different EMG channels. The method is suitable for analysis of monopolar signals and has been evaluated using high density voluntary surface EMG signals (Huang et al., 2022), but not on electrically-evoked signals. The other method is based on Radon transform (RT), which can be used to detect linear patterns in a two-dimensional signal and has been proved useful for IZ estimation (Cescon, 2006). Although IZ estimation based on RT was mainly applied to bipolar voluntary surface EMG signals (Li et al., 2021), (Cescon, 2006), (Li et al., 2022), theoretically, the RT method can also be applied on monopolar signals for estimation of IZ location.

The usefulness of PCA and RT methods for automated estimation of IZ location was explored in the current study using monopolar M waves recorded with surface electrode arrays from the biceps brachii (BB) muscles. The performance of automatic IZ detection was compared with manual detection based on visual inspection of the M waves. The objective was to provide an alternative approach to voluntary contraction for reliable and automatic estimation of muscle IZ.

## 2 Methods

### 2.1 Experiment

#### 2.1.1 Participants and consent

Nine healthy male subjects (mean ± SD, 28.9 ± 4.8 years) without a history of neuromuscular or musculoskeletal disorders participated in the study. They were well informed of the experimental procedures, including possible risks and discomforts. All subjects gave written informed consent approved by the ethics committee of Guangdong Work Injury Rehabilitation Center (Guangzhou, China).

#### 2.1.2 Experiment protocols

**FIGURE 1:**
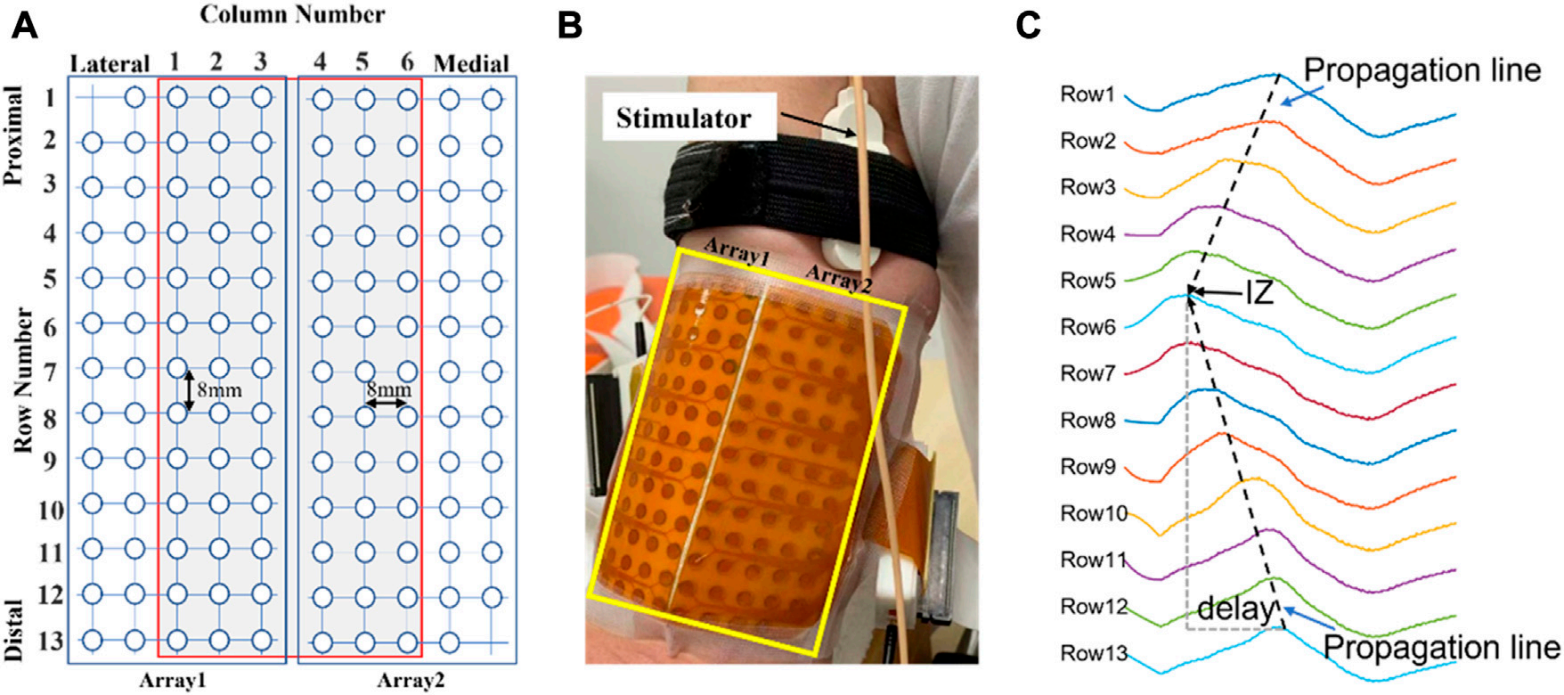
**(A)**: Schematic representation of the two adhesive 2D matrices for recording experimental signals. **(B)**: High-density electrode array recording with columns positioned parallel to the muscle fiber direction. **(C)**: An example of monopolar M waves of 13 channels in one of the columns from a representative subject. The IZ is located close to row 6. Delay: time interval of the waveforms travelling distally from the IZ.

Two high density channel arrays (ELSCH064NM2, Bioelettronica, Torino, Italy) were placed parallel to the muscle fiber direction over the lateral side (Array 1) and the medial side (Array 2) of the BB after skin preparation and fixed with elastic straps (Figure 1A). Each channel matrix consists of 64 channels with an 8 mm inter electrode distance (IED) arranged in a grid of 5 columns by 13 rows (one column contained only 12 channels). A ground electrode was placed at the elbow. A constant-current stimulator (DS7A, Digitimer, Herthfordshire, UK) and standard bar electrode (3 cm inter-electrode spacing) were used to evoke BB M-waves. The bar electrode was placed over the musculocutaneous nerve at the proximal medial side of the BB (Figure 1B). Single pulses of 1 ms duration were applied every 5 s as the current intensity was increased until the maximal M-wave was recorded. The M waves were recorded by a signal amplifier (100x) in monopolar configuration (EMG-USB2, sampling frequency of 2048 Hz, 12-bit A/D converter, Bioelettronica, Torino, Italy).

### 2.2 Detection of muscle IZ from monopolar M waves

#### 2.2.1 Muscle IZ estimation based on PCA

The rationale for using the 2nd principal component coefficients derived from PCA for IZ estimation was explained in detail (Huang et al., 2022). Briefly, PCA performs the eigen decomposition on the covariance matrix Σ of the standardized (zero mean, and unit variance) electrode array EMG signals *X* (M-by-N matrix, N samples and M channels), which is a M × M matrix where each element represents the covariance between two channels. The elements of each eigenvector are the coefficients of each principal component. It has been proven that the 2nd principal component coefficients are related with the time delays of different channels due to signal propagation from the IZ to the two ends of a muscle (Huang et al., 2022) (Laguna et al., 2018). As illustrated in Figure 1C, the channels located near the IZ are expected to have minimum time delay. Therefore, analysis of the 2nd principal component coefficients can provide useful information pertaining to IZ location.

#### 2.2.2 Muscle IZ estimation based on RT

The procedures of the RT based IZ identification have been described in detail (Cescon, 2006). For a column of signals, starting from the first row, the RT was implemented to search for the optimal propagation lines from signals at either side of a row i (i = 1,2,3 … 13), or between row j and row j + 1 (j = 1,2,3 … 12). A total of 25 RT results were obtained. As illustrated in Figure 1C, the potentials in the spatiotemporal surface EMG signal appear along inclined lines as they travel from the IZ to the tendon regions at a certain velocity. Therefore, the IZ location can be estimated by the maximum RT result.

### 2.3 Performance evaluation

The M-waves of the two most lateral and most two medial side columns were excluded from IZ identification as they were close to margin of the muscle. This left 6 columns for IZ estimation for each subject (Figure 1A). For each M wave, stimulation artifact was identified and suppressed as described previously (Liu et al., 2014). The signal duration was 0.2 s. For the PCA-based method, the signals were standardized (zero mean and unit variance). The spline interpolation was applied to the 2nd component coefficients along the rows to determine the IZ location for each column. For the RT based method, the signals were rectified. The output of the IZ detection was the channel number if the IZ was located on a specific channel or the average of neighboring channels if the IZ was located between two channels. In addition, the IZ was estimated from a conventional cross correlation method applied on bipolar M wave signals constructed from monopolar signals. The identified IZs from each of the methods were compared with those estimated manually based on visual inspection of the M waves by at least two experienced investigators. These investigators reached an agreement on IZ location prior to automated processing. The IZ location from manual inspection was used as the reference for quantifying the performance of the automated methods.

## 3 Results

**FIGURE 2:**
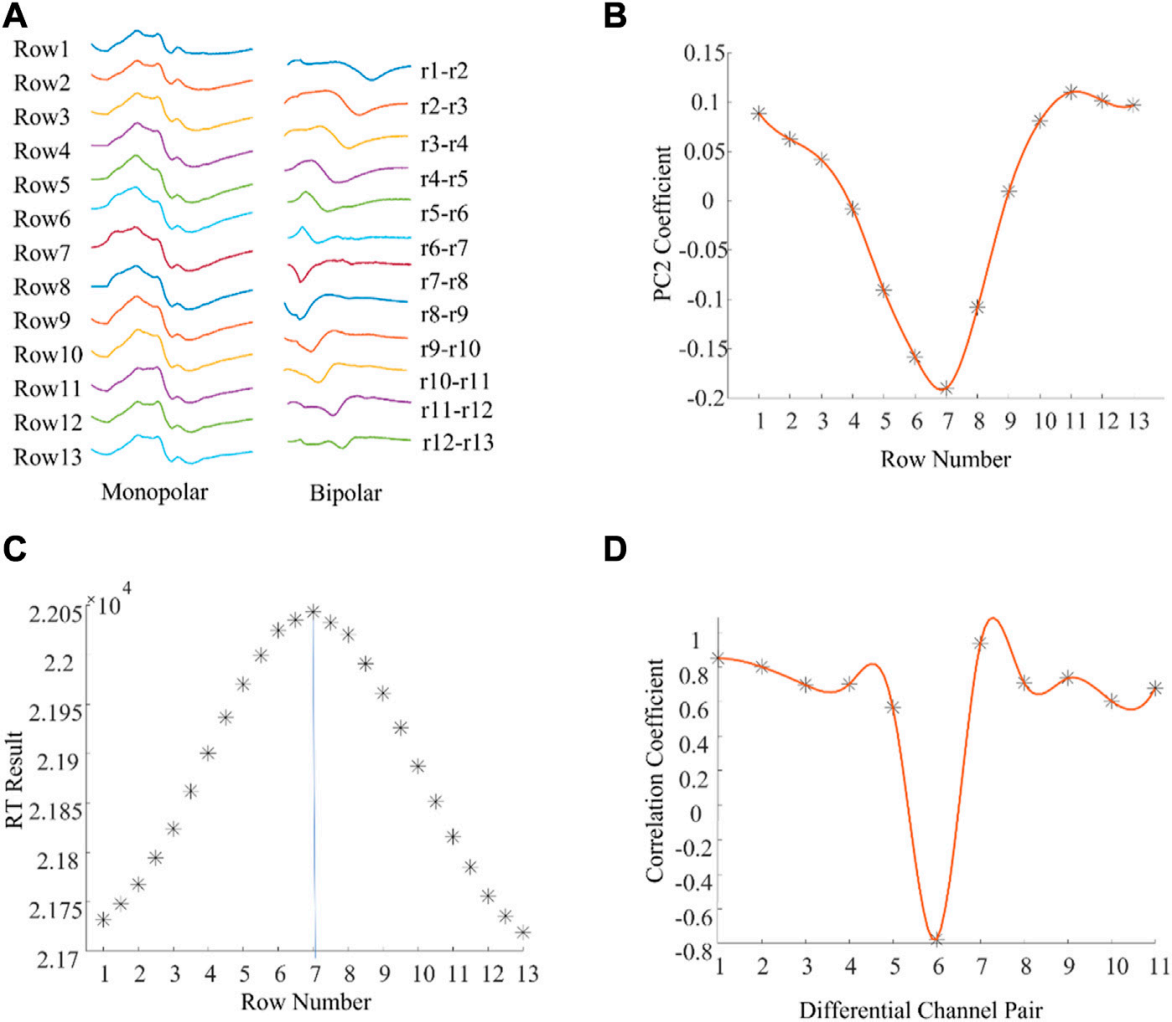
An example of muscle IZ estimation from experimental high density M waves where different methods reached the same results. **(A)**: A column of M waves of a tested subject. **(B)**: PCA based IZ estimation: the minimum coefficient was located at row 7 and the coefficients gradually increased along the fiber direction. **(C)**: RT based IZ estimation: the distribution of RT results across all the rows, and between rows. **(D)**: Cross correlation based IZ estimation: the distribution of the correlation coefficients between adjacent bipolar signals.

Figure 2A shows one column of M waves in both monopolar and bipolar configurations for a single subject. Visually, the IZ was located near the channel at row 7. Figure 2B shows spatial distribution of the 2nd component coefficients. Notice that the position of the smallest coefficients was at row 7. The RT method also identified the IZ at row 7 (Figure 2C). The minimum correlation coefficient was between bipolar pair row6—row7 and row 7—row8 (Figure 2D), which also indicated that the IZ was located at row 7 based on the monopolar configuration. These results reveal that IZ location was similar across the three methods.

**FIGURE 3:**
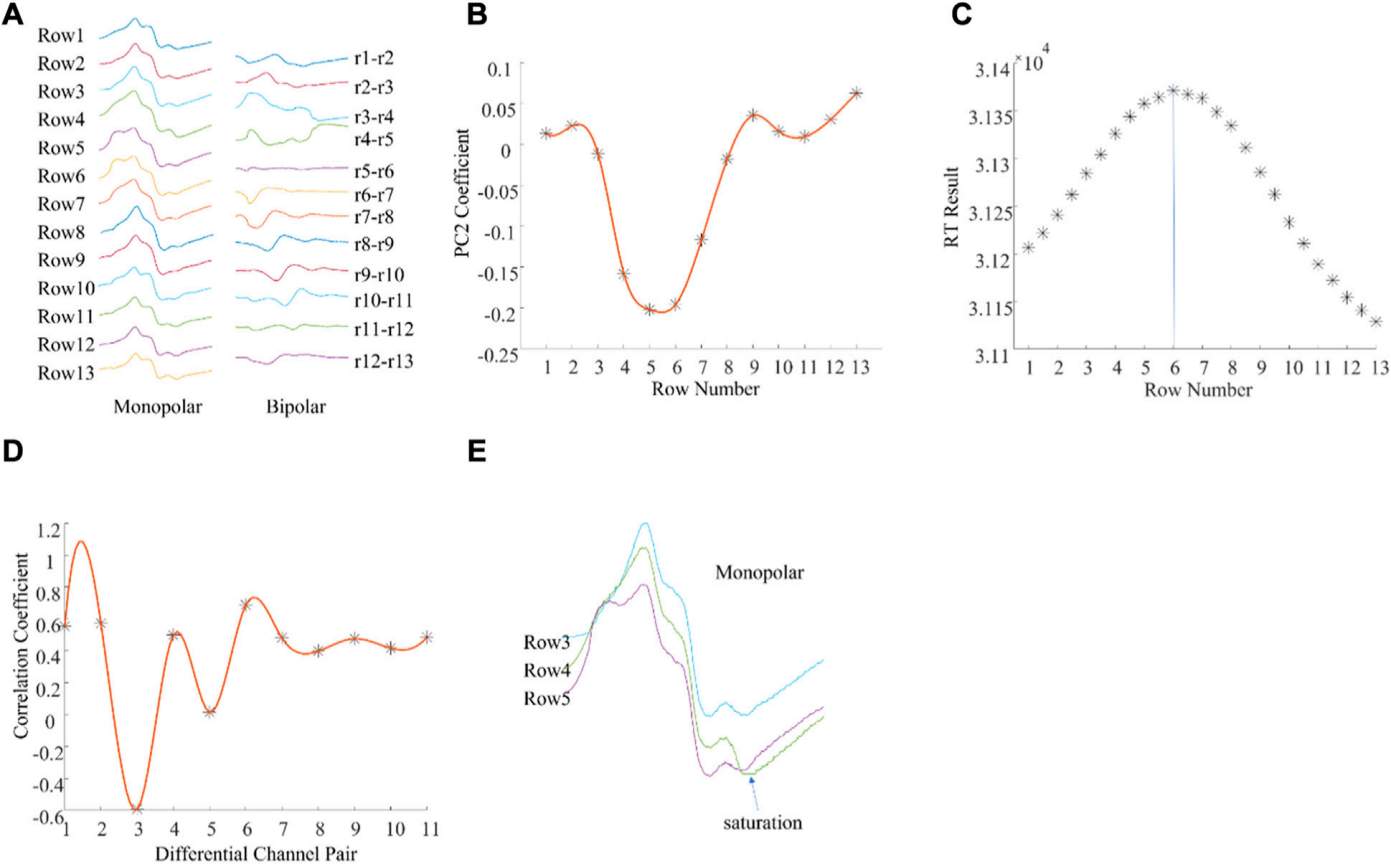
An example of muscle IZ estimation from experimental high density M waves where different methods produced different results. **(A)**: A column of M waves of a single subject. **(B)**: PCA based IZ estimation; **(C)**: RT based IZ estimation; **(D)**: Cross correlation based IZ estimation; **(E)**: Enlarged view of the monopolar M waves from rows 3, 4, and 5. See text for details.

There were also examples where the different methods produced different IZ locations (Figure 3). Figure 3A shows a column of M waves in both monopolar and bipolar configurations from a different subject. The IZ was located between row 5 and row 6 according to the 2nd principal component coefficients (Figure 3B) and at row 6 according to the maximum RT (Figure 3C). In contrast, the IZ was located at row 4 (between bipolar pair row 3—row 4, and row 4—row 5) according to the minimum correlation coefficient (Figure 3D). To explore possible reasons for the different estimates, the associated M waves were visually examined. As shown in Figure 3E, at the second phase of the M wave from row 4, there was a very short segment of saturation, which caused an artificial phase reversal between the bipolar pair row3—row4, and row4—row5, leading to misidentification of the IZ. Visual inspection of the M waves revealed that the IZ was located between row 5 and row 6. This was also confirmed from the differential signals as the amplitude between row 5 and row 6 was close to 0.

In total, 54 columns of experimental signals were processed for IZ estimation. Among them, 45 IZs (83%) estimated from the PCA, 34 IZs (63%) from the RT, and 30 IZs (56%) from the cross correlation were the same as estimations based on visual inspection. Compared with the visual estimations (reference IZs), the mean difference in estimated IZ location was 0.12 ± 0.28 IED for PCA, 0.33 ± 0.41 IED for RT, and 0.39 ± 0.74 IED for cross correlation.

## 4 Discussion

The ability to estimate muscle IZ through high density surface EMG signals using linear or 2-dimensional electrode arrays may be important clinically. A typical application is to guide BTX injection as close as possible to the IZ for spasticity treatment (Lapatki et al., 2011), (Kaymak et al., 2018). Voluntary contraction and electrical stimulation of the motor nerve are two common ways to generate surface EMG signals. The advantage of using electrical stimulation for IZ estimation is that it can be applied in patients who are paralyzed or lack the necessary voluntary control. M waves are commonly recorded in a monopolar mode, which provides informative content of action potential generation, propagation, and extinction (Rodriguez-Falces and Place, 2018). Compared with a differential configuration, monopolar recording can capture EMG signals from a larger muscle volume. The loss of M wave signal due to phase cancellation was more pronounced in bipolar than monopolar recording (Tucker and Türker, 2005). Although M waves provide a valuable signal source, its application for muscle IZ estimation has been rarely explored in the literature.

The current study examined two methods of estimating IZ location (PCA and RT) from monopolar M waves of the BB muscles. The BB was chosen as it is often affected by spasticity in patients with neurological disorders and is thus often a target muscle for treatment. Compared with manual IZ detection by an experienced investigator, the PCA based method achieved more consistent performance than one based on RT. PCA and RT use different computational approaches for IZ estimation. In PCA, a simplified time misaligned data model shows that the 2nd principal component coefficients are linearly related with the time delay of different channels (Laguna et al., 2018). Therefore, the 2nd principal component can be used for IZ estimation. The rationale of RT is that it can be used to measure the projections of the line-scan image at a range of angles and determine the propagation of waveforms. When the RT is applied for IZ estimation, it is assumed that waveforms propagate at a constant velocity on both sides of the IZ (Cescon, 2006). However, this is not always the case experimentally, as illustrated from examples of the 2^nd^ principal component coefficients distributions (Figures 2, 3). This might be one reason that the performance of RT is not as consistent as PCA.

The IZ was also estimated from cross correlation analysis applied to bipolar M waves constructed from the monopolar signals, and its performance was the least consistent relative to the visual inspected IZ. In the correlation coefficient method, if one monopolar channel is of poor signal quality, the constructed bipolar configuration may be affected leading to errors in IZ location (Figure 3). This was also demonstrated in our previous study (Huang et al., 2022).

The experimental data sets used for evaluating IZ estimation performance were limited to the BB of healthy subjects. Recordings from other muscles in the future is desirable. It would be clinically relevant to test patients with neurological disorders such as stroke. The IED of the electrode array used in this study was 8 mm. This limited the spatial resolution for IZ detection, but can be increased by using a smaller IED. In addition, this study only considered a single IZ in a muscle. The effects of possible multiple IZs on recorded M waves needs further investigation, as they may compromise accuracy of the estimated IZ (Piccoli et al., 2014), (Huang et al., 2021) (Lateva et al., 2010).

In summary, the current study explored the feasibility of estimating IZ using monopolar high density BB M waves. The PCA based method was able to automatically detect muscle IZs from monopolar M waves, demonstrating a performance most consistent with manual detection by human operators. The findings provide an alternative approach to voluntary contractions for estimating the IZ, which has practical clinical value for patients with compromised ability to voluntarily activate their skeletal musculature.

## Data Availability

The raw data supporting the conclusion of this article will be made available by the authors, without undue reservation.
